# Selective and Efficient Generation of *ortho*-Brominated *para*-Substituted Phenols in ACS-Grade Methanol

**DOI:** 10.3390/molecules21010088

**Published:** 2016-01-13

**Authors:** David Georgiev, Bartholomeus W. H. Saes, Heather J. Johnston, Sarah K. Boys, Alan Healy, Alison N. Hulme

**Affiliations:** EaStCHEM School of Chemistry, The University of Edinburgh, David Brewster Road, Edinburgh EH9 3FJ, UK; D.Georgiev@sms.ed.ac.uk (D.G.); bartsaes@live.nl (B.W.H.S.); hjohnst2@exseed.ed.ac.uk (H.J.J.); boys.sarah@gmail.com (S.K.B.); alan.healy@yale.edu (A.H.)

**Keywords:** *ortho*-bromination, ACS-grade methanol, NBS

## Abstract

The mono *ortho*-bromination of phenolic building blocks by NBS has been achieved in short reaction times (15–20 min) using ACS-grade methanol as a solvent. The reactions can be conducted on phenol, naphthol and biphenol substrates, giving yields of >86% on gram scale. Excellent selectivity for the desired mono *ortho*-brominated products is achieved in the presence of 10 mol % *para*-TsOH, and the reaction is shown to be tolerant of a range of substituents, including CH_3_, F, and NHBoc.

## 1. Introduction

**Figure 1:**
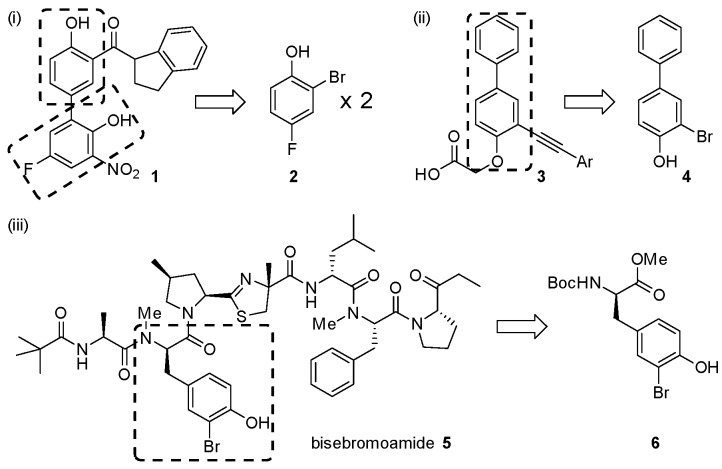
Recent applications of mono *ortho*-brominated phenols to the synthesis of biologically active targets.

Brominated phenols and their derivatives constitute important building blocks for a range of synthetic targets (Figure 1), with recent examples of pharmaceutical interest including: (i) synthesis of aryl 1-indanylketone inhibitors of the human peptidyl prolyl *cis*/*trans* isomerase Pin1, such as **1**, using a domino coupling reaction starting from the methylated derivative of 2-bromo-4-fluorophenol **2** [1]; (ii) synthesis of alkynylphenoxyacetic acid CRTH2 (DP2) receptor antagonists, such as library members **3**, by Sonogashira coupling to the *tert*-butyl protected phenoxyacetic acid derivative of 3-bromo-[1,1′-biphenyl]-4-ol **4** [2]; and (iii) synthesis of the cytotoxic peptidic marine natural product bisebromoamide **5** [3], through modification and peptide coupling of brominated D-tyrosine derivatives such as **6** [4,5].

**Scheme 1:**
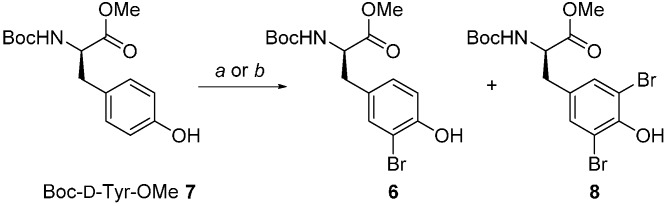
Building block synthesis for the marine natural product bisebromoamide **5**. *Reagents and Conditions*: (**a**) NBS (100 mol %), In(OTf)_3_ (10 mol %), MeCN, rt, 10 min; (**b**) NBS (100 mol %), *p*TsOH (10 mol %), EtOAc, rt, 30 h.

As part of our own synthetic efforts directed towards the efficient solid-phase synthesis of bisebromoamide **5** [6,7], gram-scale syntheses of each of the component building blocks was required [8,9]. For the bromotyrosine derivative a number of recently published methods for the *N*-bromosuccinimide (NBS) promoted mono-*ortho*-bromination reaction were surveyed with a view to applying them to the amino acid derivative Boc-d-Tyr-OMe (**7**→**6**, Scheme 1). Of particular concern was minimization of over-bromination which results in the dibromo derivative **8**, removal of which by chromatography greatly reduced synthetic efficiency.

Initial attempts to brominate Boc-d-Tyr-OMe **7** focused on indium(III) triflate catalysis of the reaction of NBS in acetonitrile [10]. However, the major product of the reaction was found to be the dibrominated material **8**, even when the reaction was conducted in the dark and at reduced temperature (0 °C). The Chhattise group have demonstrated rapid (<10 min) photochemical bromination of aromatic compounds (including *para-*substituted phenols) under UV-vis irradiation at ambient temperatures [11]. Reaction of Boc-d-Tyr-OMe **7** with one equivalent of NBS in ethyl acetate under UV-vis irradiation (365 nm; 6 W, F6T5/BL lamp) resulted in rapid consumption of the starting material, but again gave predominantly the undesired dibrominated product **8**.

In an attempt to minimize the dibromination reaction, attention then shifted to the *p*-toluene-sulfonic acid (*p*TsOH)-mediated NBS bromination reaction [12,13]. Leykajarakul and co-workers have proposed a mechanism for this reaction in which the *p*TsOH conjugates to the phenolic alcohol directing bromination to the *para* position, or in the case of *para*-substituted phenols to give selective mono *ortho*-bromination [11,13,14]. When Boc-d-Tyr-OMe **7** was reacted with NBS in the presence of *p*TsOH (10 mol %) in ethyl acetate a moderate yield (58%) of the mono-brominated material **6** was obtained after 30 h reaction time. UV-vis irradiation (365 nm; 6 W, F6T5/BL lamp) of this reaction gave full conversion to the desired monobrominated product **6** in times ranging from 40 min to 4 h, depending on the reaction scale and light source used. However, it was suspected that the extended reaction times also led to UV-mediated debromination of the desired product, complicating the reaction still further. For these reasons, reproducible reaction conditions which were readily amenable to scale-up were sought. Flow chemistry, and in particular photochemical flow chemistry with its short path lengths allowing efficient irradiation, readily controlled residence times and scaleability [15,16,17,18,19], was initially considered as a potential solution.

## 2. Results and Discussion

### 2.1. Investigation of the Mono ortho-Bromination Flow Reaction

Investigation of the mono *ortho*-bromination reaction by NBS in the presence of *p*TsOH (10 mol %) under flow conditions was carried out using a UV photoreactor (365–366 nm; 125 W, Hg lamp) based on those reported by the Booker-Milburn and Seeberger groups [16,17,18,19]. The initial batch reactions had been conducted in ethyl acetate, but NBS shows only limited solubility in this solvent making it incompatible with a flow modality for the reaction. For this reason the flow reaction was explored in both acetonitrile and methanol; two solvents which are commonly used in bromination reactions on other classes of substrates. Although higher concentrations of NBS could be achieved in acetonitrile than in methanol (0.5 M and 0.1 M respectively) which would lead to increased production rates in flow, the reactions in acetonitrile were noticeably slower with longer residence times required in the UV photoreactor to achieve full conversion. Indeed, across a range of substrates the observed rates of reaction in methanol were so fast that it led us to suspect that exposure to the UV light source was not required. This was confirmed by reactions conducted under standard flow conditions but in the absence of UV light (the so-called “dark reaction”), in which reactive substrates showed full conversion to the mono *ortho*-brominated products in methanol, but only minimal conversion in acetonitrile. These observations led us to look more closely at the NBS-mediated mono *ortho*-bromination reaction in methanol.

### 2.2. Investigation of Batch Reaction Conditions Using ACS-Grade Methanol

There are very few reports of *ortho*-bromination reactions of phenols conducted in methanol; and those which have been reported to give mono selectivity rely on blocking of the second *ortho* position to achieve this [14]. However, a similar dramatic rate acceleration for the NBS-mediated bromination of the polyalkylated aromatic durene in the presence of *p*TsOH has been reported when this reaction was conducted in methanol rather than acetonitrile, or ethyl acetate [20]. The facile chlorination of aromatic substrates in water at 40 °C using a combination of NCS/NaCl reagents in the presence of *p*TsOH has also been reported [21,22]. It has been proposed that under these polar protic conditions the *p*TsOH accelerates the reaction through protonation of the *N*-halosuccinimide to provide a more reactive electrophile [20,21].

In investigating the selective mono *ortho*-bromination reaction by NBS in the presence of *p*TsOH in methanol, the reaction of *p*-cresol **9** (Table 1) was used as a test substrate, as it was both readily available and the reaction to give the desired mono *ortho*-brominated product **10** (and the undesired dibrominated product **11**) could be easily followed by HPLC (SI Figure S1). The *p*-cresol **9** was premixed with *p*TsOH prior to addition of NBS as this has been shown to give better selectivity for mono *ortho*-bromination [12]. HPLC analysis of samples taken directly from the reaction mixture at timed intervals rapidly established that the batch reaction of **9** in methanol reached completion in under 5 min, even in the absence of light. When the reaction was carried out with NBS addition in a single portion at the beginning of the reaction, bromination of *p*-cresol **9** gave conversion to **9**:**10**:**11** in a ratio of 6:87:7 (Table 1, entry 1).

**Table 1:** Optimization of batch conditions for the mono *ortho*-bromination reaction. 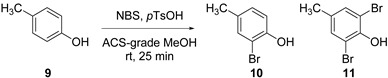

Entry	NBS (mol %)	*p*TsOH (mol %)	Ratio (Relative % at 285 nm)			Isolated Yield 10 (%)
			9	10	11	
1 ^a,b^	100	10	6	87	7	--
2 ^b^	100	10	5	93	2	--
3	100	10	3	94	3	92%
4	110	10	0	88	12	86%
5	100	0	15	77	8	74%
6	100	20	3	93	4	90%

^a^ NBS added as a single portion at the start of the reaction and then reaction stirred for 25 min; ^b^ Reaction conducted in dry methanol.

When the bulk concentration of NBS was reduced through its controlled addition in solution by cannula, an overall increase in conversion to the desired mono *ortho*-brominated product **10** was observed (Table 1, entry 2). The reaction was found to be tolerant of moisture (Table 1, entries 2 and 3); when the reaction was conducted in ACS-grade methanol, under ambient conditions (air and room temperature) an excellent isolated yield of **10** was achieved and no significant change in the ratio of products was observed. Selectivity for the desired mono *ortho*-brominated product was shown to depend heavily on the number of equivalents of NBS used (Table 1, entries 3 and 4), but also to be influenced by the presence of *p*TsOH (Table 1, entries 5 and 6) as previously reported [12,13].

Optimum conditions involved pre-mixing the substrate and *p*TsOH (10 mol %) in a minimal amount of ACS-grade methanol and controlled addition of a solution of NBS (100 mol %) as a 0.1 M solution in methanol over 20 min, then stirring for a further 5 min, at ambient temperature (~20 °C) under air; giving a ratio of **9**:**10**:**11** of 3:94:3 and an isolated yield of **9** of 92%. These conditions were then successfully applied to a range of substrates on gram scale as shown in Table 2.

**Table 2:** Gram scale mono *ortho*-bromination reaction in ACS-grade methanol.

Entry	Starting Material ^a^	Major Product	Ratio (SM:mono:di) ^b^	Isolated Yield ^c^ (%)
1	*p*-Cresol **9**	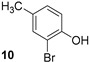	1:95:4	92
2	4-Fluorophenol	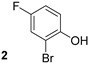	4:89:7	86
3	Vanillin	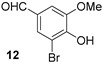	5:95:--	90
4	2-Naphthol	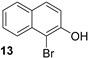	0:99:1	98
5	4-*tert*-Butylphenol	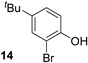	2:92:6	90
6	[1,1′-Biphenyl]-4-ol	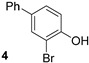	4:90:6	89
7	3′-Fluoro-6′-methoxy-[1,1′-biphenyl]-4-ol	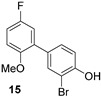	4:92:4	90
8	Boc-Tyr-OMe *ent*-**7**	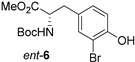	2:94:4	89

^a^ Method A: NBS (100 mol %), *p*TsOH (10 mol %), MeOH, rt, 25 min; ^b^ Ratio SM:mono:di = starting material:mono *ortho*-brominated:di *ortho*-brominated; ^c^ Major product.

Phenol derivatives with alkyl and aryl *para*-substituents (entries 1, 5–7, Table 2) and Boc-Tyr-OMe (*ent*-**7**, entry 8, Table 2) were shown to give high selectivity for the mono *ortho*-brominated products under these conditions, facilitating separation to give excellent yields of the major product. Vanillin and 2-naphthol (entries 3 and 4, Table 2), both known to react readily to bromination, also gave excellent conversion. Electron withdrawing *para*-substituents (entry 2, Table 2, and *p*-CF_3_ not shown) generally induced poorer selectivity for the mono *ortho*-brominated products resulting in somewhat reduced isolated yields of the major products.

## 3. Experimental Section

### 3.1. General Information

ACS-grade methanol was obtained from Fischer Scientific and was used without further purification or drying; all other chemicals were used as obtained from the supplier unless otherwise stated. ^1^H-, ^13^C- and ^19^F-NMR spectra were obtained on an AVA 500 or a PRO 500 instrument (Bruker, Coventry, UK) using TMS as a reference and residual solvent as an internal standard. The data are presented as follows: chemical shift (in ppm on the δ scale relative to δ_TMS_ = 0), integration, multiplicity, coupling constant and interpretation. Electron ionisation (EI) mass spectra were obtained on a MS50TC mass spectrometer (Kratos, Manchester, UK). Analytical Reverse Phase HPLC (RP-HPLC) was conducted on a Waters^®^ 600 (100 µL) system (Waters, Elstree, UK) using a 717plus autosampler and 996 PDA detector (190 to 800 nm) equipped with a Phenomenex^®^ Luna C18(2) 5 µm column (i.d. 4.6 mm, length 300 mm). A binary solvent system was used A = water (0.1% TFA), B = MeCN (0.1% TFA) at a flow rate of 1.00 mL·min^−1^; and the column was maintained at 30 ± 1 °C. The elution program was a linear gradient from 0 min (95A:5B) to 30 min (5A:95B), isocratic from 30 min to 35 min (5A:95B), before recovery of the initial conditions over 5 min and equilibration over 10 min, giving a total run time of 50 min. Melting points were determined on an Electrothermal Melting Point apparatus (Gallenkamp, Loughborough, UK) and are uncorrected. Optical rotations were performed on a POLAAR 20 polarimeter (Optical Activity, Ramsey, UK).

### 3.2. General Procedure for Batch Reaction Conditions in ACS-Grade Methanol

A solution of the starting material (~10 mmol) and *p*TsOH (10 mol %) in MeOH (1.0 mL per mmol starting material) was stirred for 10 min, then a solution of NBS (100 mol %; recrystallized from H_2_O) in MeOH (0.1 M) was added dropwise over 20 min from a foiled reaction flask. The reaction mixture was stirred for a further 5 min and then concentrated *in vacuo*. The resultant residue was purified using column chromatography (CH_2_Cl_2_, or 1% MeOH in CH_2_Cl_2_).

### 3.3. Characterization of Products

*2-Bromo-4-methylphenol* (**10**) [23]: 10.1 mmol; 1.73 g (92%); Rt = 24.3 min; ^1^H-NMR δ (500 MHz, CDCl_3_) 7.30 (1H, br s, Ar*H*), 7.04 (1H, br d, *J* = 8.2 Hz, Ar*H*), 6.94 (1H, d, *J* = 8.2 Hz, Ar*H*), 5.43 (1H, s, O*H*), 2.30 (3H, s, OC*H*_3_); ^13^C-NMR δ (126 MHz, CDCl_3_) 150.0 (C), 132.2 (CH), 131.4 (C), 129.8 (CH), 115.8 (CH), 109.9 (C), 20.2 (CH_3_); *m*/*z* (EI) 188 (^81^BrM^+^, 52%), 186 (^79^BrM^+^, 54), 107 (100), 77 (35).

*2,6-Dibromo-4-methylphenol* (**11**) [24]: Rt = 27.6 min; mp 47–49 °C, lit [24] 49–51 °C; ^1^H-NMR δ (500 MHz, CDCl_3_) 7.26 (2H, br s, Ar*H*), 5.70 (1H, s, O*H*), 2.25 (3H, s, OC*H_3_*); ^13^C-NMR δ (126 MHz, CDCl_3_) 147.1 (C), 132.4 (2 × CH and C), 109.4 (2 × C), 20.0 (CH_3_); *m*/*z* (EI) 268 (^81^Br^81^BrM^+^, 46%), 266 (^81^Br^79^BrM^+^, 100), 264 (^79^Br^79^BrM^+^, 48), 187 (39),185 (41).

*2-Bromo-4-fluorophenol* (**2**) [23]: 10.3 mmol; 1.70 g (86%); Rt = 23.1 min; ^1^H-NMR δ (500 MHz, CDCl_3_) 7.21 (1H, ddd, *J* = 7.7, 2.5 and 0.7 Hz Ar*H*), 7.00–6.93 (2H, m, 2 × Ar*H*), 5.32 (1H, s, O*H*); ^13^C-NMR δ (126 MHz, CDCl_3_) 156.4 (d, ^1^*J*_CF_ = 242 Hz, C), 148.9 (d, ^4^*J*_CF_ = 3 Hz, CH), 118.7 (d, ^2^*J*_CF_ = 26 Hz, C), 116.3 (d, ^3^*J*_CF_ = 8 Hz, CH), 116.0 (d, ^2^*J*_CF_ = 23 Hz, CH), 109.5 (d, ^3^*J*_CF_ = 10 Hz, C); ^19^F-NMR δ (471 MHz, CDCl_3_) −121.97 (td, *J* = 7.7 and 5.4 Hz); *m*/*z* (EI) 192 (^81^BrM^+^, 98%), 190 (^79^BrM^+^, 100), 82 (43), 83 (27).

*3-Bromo-4-hydroxy-5-methoxybenzaldehyde* (**12**) [25]: 9.09 mmol; 1.89 g (90%); Rt = 21.4 min; mp 160–162 °C, lit [25] 162–164 °C, EtOH; ^1^H-NMR δ (500 MHz, DMSO-*d*_6_) 10.72 (1H, s, O*H*), 9.79 (1H, s, C*H*O), 7.73 (1H, d, *J* = 1.8 Hz, Ar*H*), 7.43 (1H, d, *J* = 1.8 Hz, Ar*H*), 3.92 (3H, s, OC*H*_3_); ^13^C-NMR δ (126 MHz, DMSO-*d*_6_) 190.9 (CH), 150.3 (C), 149.1 (C), 129.4 (C), 129.2 (CH), 110.1 (CH), 109.7 (C), 56.8 (CH_3_); *m*/*z* (EI) 232 (^81^BrM^+^, 94%), 231 (86) 230 (^79^BrM^+^, 100), 229 (95).

*1-Bromo-2-naphthol* (**13**) [23]: 9.05 mmol; 1.98 g (98%); Rt = 26.4 min; mp 80–82 °C, lit [23] 82–83 °C; ^1^H-NMR δ (500 MHz, CDCl_3_) 8.06 (1H, br d, *J* = 8.4 Hz, Ar*H*), 7.81 (1H, br d, *J* = 8.1 Hz, Ar*H*), 7.77 (1H, d, *J* = 8.8 Hz, Ar*H*), 7.60 (1H, ddd, *J* = 8.4, 6.9 and 1.2 Hz, Ar*H*), 7.42 (1H, ddd, *J* = 8.1, 6.9 and 1.0 Hz, Ar*H*), 7.30 (1H, d, *J* = 8.8 Hz, Ar*H*), 5.94 (1H, s, O*H*); ^13^C-NMR δ (126 MHz, CDCl_3_) 150.6 (C), 132.3 (C), 129.7 (C), 129.4 (CH), 128.2 (CH), 127.9 (CH), 125.3 (CH), 124.2 (CH), 117.2 (CH), 106.2 (C); *m*/*z* (EI) 224 (^81^BrM^+^, 98%), 222 (^79^BrM^+^, 100), 115 (26), 114 (36).

*2-Bromo-4-tert-butylphenol* (**14**) [26]: 10.1 mmol; 2.07 g (90%); Rt = 27.6 min; ^1^H-NMR δ (500 MHz, CDCl_3_) 7.48 (1H, d, *J* = 2.3 Hz, Ar*H*), 7.27 (1H, dd, *J* = 8.5 and 2.3 Hz, Ar*H*), 6.99 (1H, d, *J* = 8.5 Hz, Ar*H*), 5.39 (1H, s, O*H*), 1.32 (9H, s, C(C*H*_3_)_3_); ^13^C-NMR δ (126 MHz, CDCl_3_) 149.9 (C), 145.1 (C), 128.8 (CH), 126.3 (CH), 115.6 (CH), 109.9 (C), 34.2 (C), 31.4 (3 × CH_3_); *m*/*z* (EI) 230 (^81^BrM^+^, 76%), 228 (^79^BrM^+^, 74), 215 (100), 213 (86), 134 (87).

*3-Bromo-[1,1′-biphenyl]-4-ol* (**4**) [27]: 10.0 mmol; 2.22 g (89%); Rt = 24.2 min; mp 92–94 °C, lit [27] 94–95 °C; ^1^H-NMR δ (500 MHz, CDCl_3_) 7.70 (1H, d, *J* = 2.2 Hz), 7.56–7.48 (2H, m), 7.46 (1H, dd, *J* = 8.4 and 2.2, Hz), 7.44–7.39 (2H, m), 7.37–7.29 (1H, m), 7.09 (1H, d, *J* = 8.4 Hz), 5.52 (1H, s, O*H*); ^13^C-NMR δ (126 MHz, CDCl_3_) 151.7 (C), 139.5 (C), 135.4 (C), 130.5 (CH), 128.9 (2 × CH), 128.0 (CH), 127.3 (CH), 126.8 (2 × CH), 116.3 (CH), 110.7 (C); *m*/*z* (EI) 250 (^81^BrM^+^, 100%), 248 (^79^BrM^+^, 100), 139 (38), 86 (58), 84 (99).

*3-Bromo-3′-fluoro-6′-methoxy-[1,1′-biphenyl]-4-ol* (**15**): 3.49 mmol; 0.93 g (90%); Rt = 28.3 min; ^1^H-NMR δ (500 MHz, CDCl_3_) 7.67 (1H, d, *J* = 2.1 Hz, Ar*H*), 7.41 (1H, dd, *J* = 8.4 and 2.1 Hz, Ar*H*), 7.09 (1H, d, *J* = 8.4 Hz, Ar*H*), 7.03 (1H, ddd, *J* = 9.2, 3.1 and 0.7 Hz, Ar*H*), 7.03–6.98 (1H, m, Ar*H*), 6.91 (1H, ddd, *J* = 8.7, 4.5 and 0.7 Hz, Ar*H*), 5.57 (1H, s, O*H*), 3.81 (3H, s, OC*H*_3_); ^13^C-NMR δ (126 MHz, CDCl_3_) 157.1 (d, ^1^*J*_CF_ = 239 Hz, C), 152.5 (d, ^4^*J*_CF_ = 2 Hz, C), 151.6 (C), 132.7 (CH), 131.3 (d, ^4^*J*_CF_ = 2 Hz, C), 130.3 (CH), 130.1 (d, ^3^*J*_CF_ = 8 Hz, C), 117.1 (d, ^2^*J*_CF_ = 24 Hz, CH), 115.6 (CH), 114.4 (d, ^2^*J*_CF_ = 23 Hz, CH), 112.3 (d, ^3^*J*_CF_ = 8 Hz, CH), 109.9 (C), 56.2 (CH_3_); ^19^F-NMR δ (471 MHz, CDCl_3_) −123.79 (ddd, *J* = 9.2, 8.3 and 4.5 Hz); *m*/*z* (EI) 298 (^81^BrM^+^, 89%), 296 (^79^BrM^+^, 90), 203 (20) 202 (100); HRMS (EI) ^79^BrM^+^ found 295.9839, C_13_H_10_O_2_^79^BrF requires 295.9843.

*Methyl (S)-2-tert-butoxycarbonylamino-3-(3-bromo-4-hydroxyphenyl)propanoate* (*ent*-**6**): 5.00 mmol; 1.68 g (89%); Rt = 25.3 min; [α]_D_ = 59.0 (c 1, CHCl_3_); mp 117–119 °C; ^1^H-NMR δ (500 MHz, CDCl_3_, 323 K) 7.24 (1H, d, *J* = 2.0 Hz, Ar*H*), 6.97 (1H, dd, *J* = 8.3 and 2.0 Hz, Ar*H*), 6.90 (1H, d, *J* = 8.3 Hz, Ar*H*), 5.50 (1H, br s, O*H*), 5.01 (1H, br s, N*H*), 4.51 (1H, br s, α-C*H*), 3.71 (3H, s, OC*H_3_*), 3.04 (1H, dd, *J* = 14.0 and 5.8 Hz, C*H*_A_H_B_Ar), 2.97—2.88 (1H, m, CH_A_*H*_B_Ar), 1.43 (9H, s, C(C*H*_3_)_3_); ^13^C-NMR δ (126 MHz, CDCl_3_, 323 K) 172.2 (C), 155.2 (C), 151.8 (C), 133.0 (CH), 130.1 (CH), 129.8 (C), 116.3 (CH), 110.2 (C), 80.3 (C), 54.7 (CH), 52.3 (CH_3_), 37.5 (CH_2_), 28.4 (3 × CH_3_); *m*/*z* (ESI+, MeOH) 398 ([81BrM + Na]^+^, 100%), 396 ([79BrM + Na]^+^, 99), 374 (14) 342 (10), 340 (11); HRMS (ESI+, MeOH) [M + Na]^+^ found 396.0434, C_15_H_20_O_5_N^79^BrNa requires 396.0417.

## 4. Conclusions

In assessing the use of methanol as a carrier solvent for the mono *ortho*-bromination of phenols by NBS under flow conditions, the “dark reaction” clearly indicated that UV-vis irradiation was not a prerequisite for success. Thus batch reaction conditions were optimized to allow a range of NBS-mediated mono *ortho*-bromination reactions to be carried out in ACS-grade methanol on gram scale without UV-vis irradiation in reaction times of 25 min. The highly selective product distributions achieved meant that the desired products of these reactions were readily purified in excellent yields (86%–98%), thus providing an extremely facile route to high value medicinal chemistry building blocks. With an efficient route to the synthesis of the mono *ortho*-brominated derivative of Boc-d-Tyr-OMe in hand, the synthesis of the intriguing anti-cancer natural product, bisebromoamide, may now be tackled.

## Supplementary Materials

The following are available online at http://www.mdpi.com/1420-3049/21/1/88/s1, Figure S1: Choice of wavelength for reaction monitoring, Figure S2: ^1^H- and ^13^C-NMR spectra for compounds **15** and *ent*-**6**.
