# Supplementary material for: Selective and Efficient Generation of ortho-Brominated para-Substituted Phenols in ACS-Grade Methanol
Source: Molecules. 2016 Jan 13;21(1):88. doi: 10.3390/molecules21010088 (PMC6274440; doi:10.3390/molecules21010088)
Supplement: Supplementary file 1 [file molecules-21-00088-s001.pdf]

Supplementary Materials: Selective and Efficient Generation of *ortho*-Brominated *para*-Substituted Phenols in ACS-Grade Methanol

David Georgiev, Bartholomeus W. H. Saes, Heather J. Johnston, Sarah K. Boys, Alan Healy and Alison N. Hulme

**Page**	**Contents**
S1–S2	**Figure S1.** Choice of wavelength for reaction monitoring.
S2–S4	**Figure S2.** ^{1}H- and ^{13}C-NMR spectra for compounds **15** and *ent*-**6**.

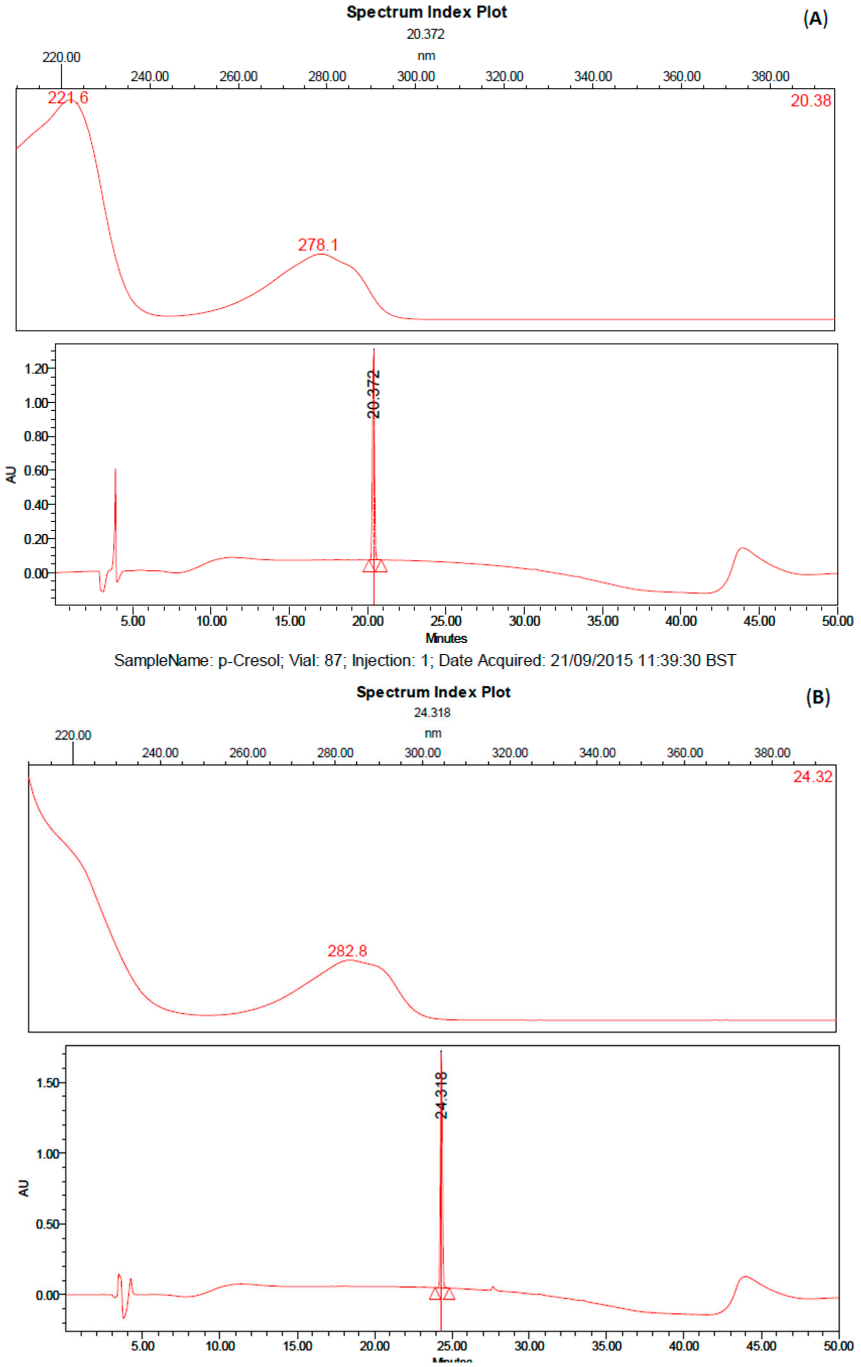

Figure S1. *Cont.*

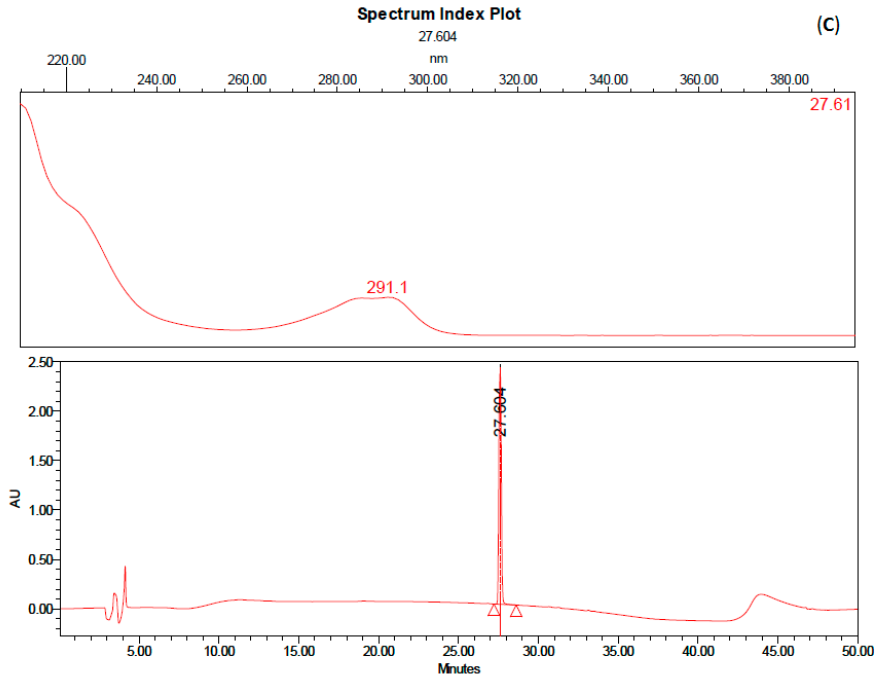

Figure S1. Choice of wavelength for reaction monitoring. (**A**). Chromatograph (lower) and PDA (upper) of *p*-cresol **9** (R_t = 20.4 min, λ_{max} = 278 nm); (**B**). Chromatograph (lower) and PDA (upper) of 2-bromo-4-methylphenol **10** (R_t = 24.3 min, λ_{max} = 283 nm); (**C**). Chromatograph (lower) and PDA (upper) of 2,4-dibromo-4-methylphenol **11** (R_t = 27.6 min, λ_{max} = 291 nm).

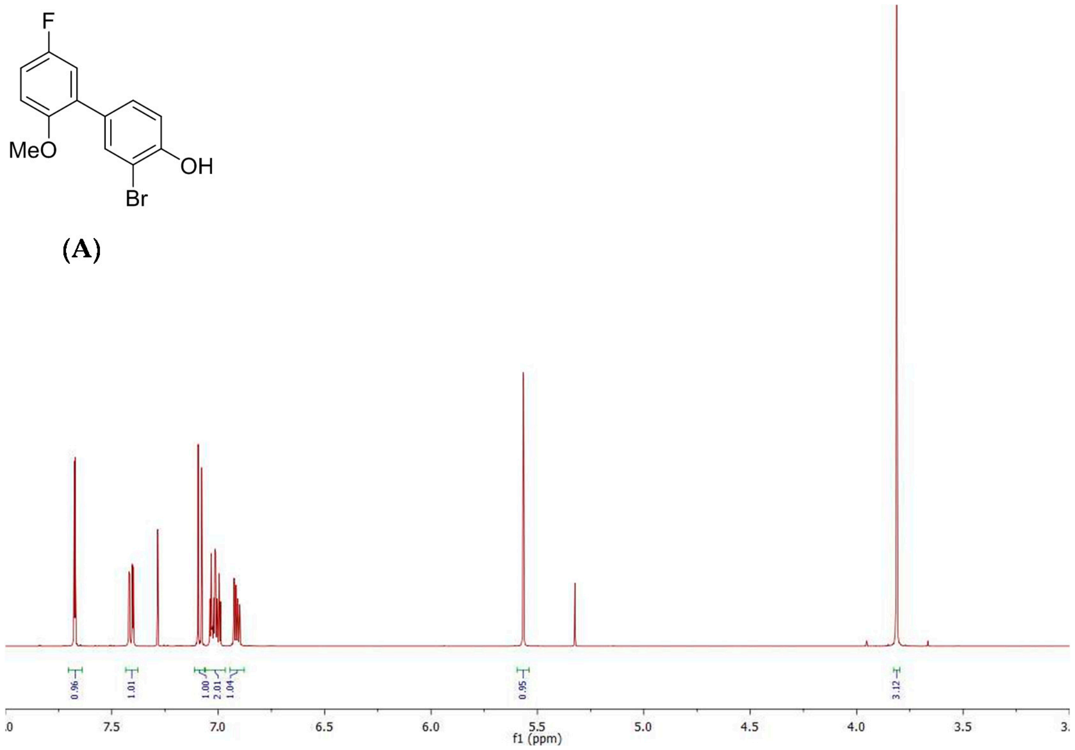

Figure S2. *Cont.*

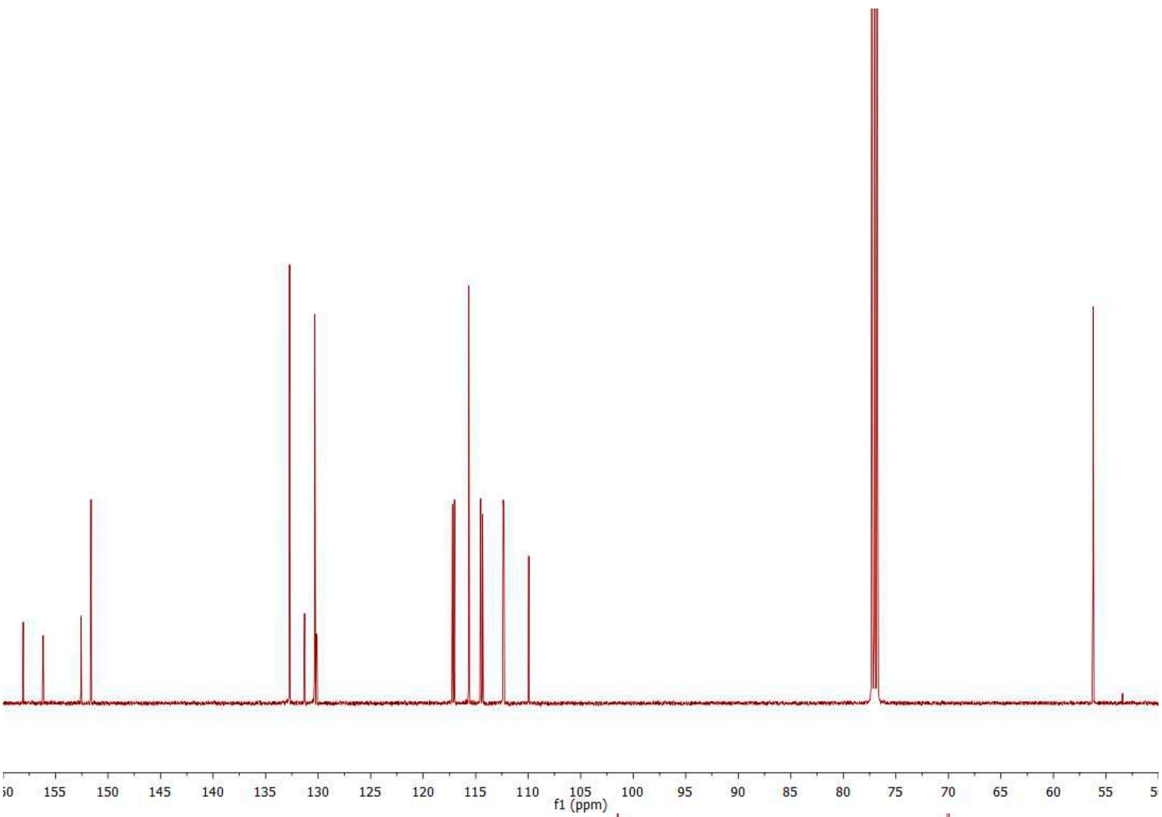

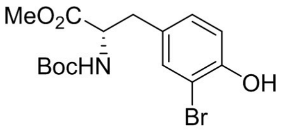

(B)

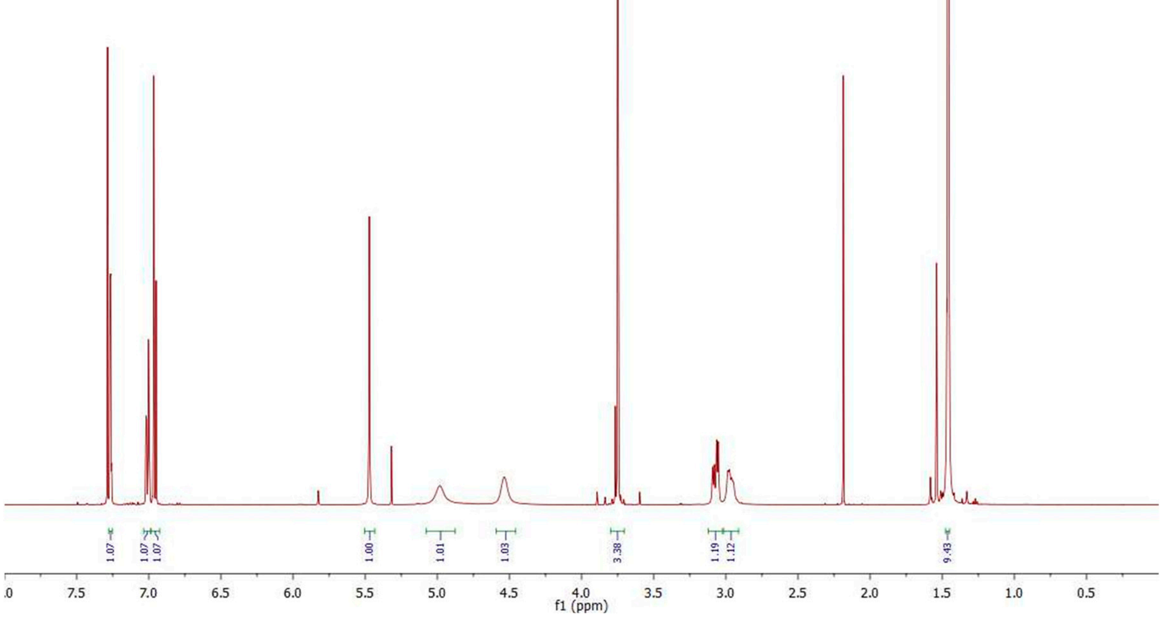

Figure S2. *Cont.*

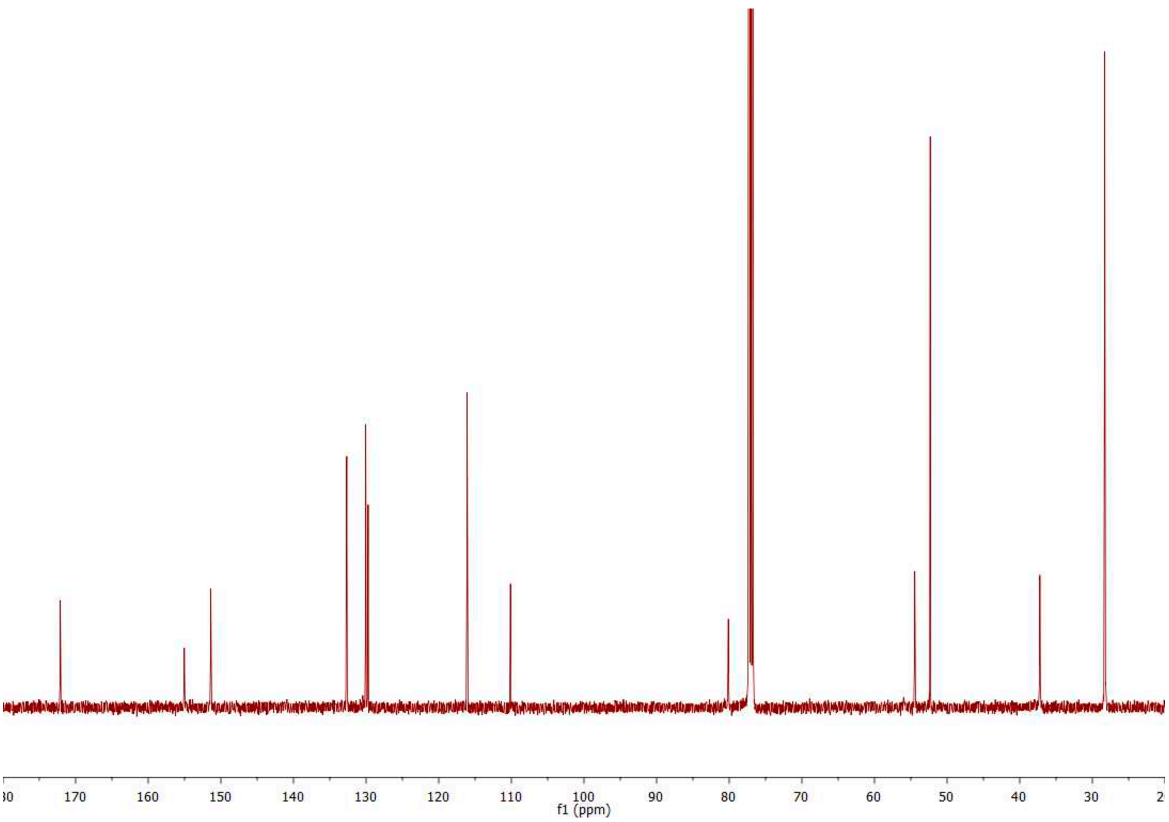

Figure S2. ^{1}H- and ^{13}C-NMR spectra for compounds **15** and *ent*-**6**. (**A**). 3-Bromo-3′-fluoro-6′-methoxy-[1,1′-biphenyl]-4-ol (**15**) ($CDCl_3$); (**B**). Methyl (*S*)-2-*tert*-butoxycarbonylamino-3-(3-bromo-4-hydroxyphenyl) propanoate (*ent*-**6**) ($CDCl_3$, 323K).
